# Increased hepatoprotective effects of the novel farnesoid X receptor agonist INT-787 versus obeticholic acid in a mouse model of nonalcoholic steatohepatitis

**DOI:** 10.1371/journal.pone.0300809

**Published:** 2024-04-25

**Authors:** Luciano Adorini, Kristoffer Rigbolt, Michael Feigh, Jonathan Roth, Mary Erickson

**Affiliations:** 1 Intercept Pharmaceuticals, Inc., San Diego, California, United States of America; 2 Gubra, Hørsholm, Denmark; University of Navarra School of Medicine and Center for Applied Medical Research (CIMA), SPAIN

## Abstract

The nuclear farnesoid X receptor (FXR), a master regulator of bile acid and metabolic homeostasis, is a key target for treatment of nonalcoholic steatohepatitis (NASH). This study compared efficacy of FXR agonists obeticholic acid (OCA) and INT-787 by liver histopathology, plasma biomarkers of liver damage, and hepatic gene expression profiles in the Amylin liver NASH (AMLN) diet–induced and biopsy-confirmed Lep^*ob/ob*^ mouse model of NASH. Lep^*ob/ob*^ mice were fed the AMLN diet for 12 weeks before liver biopsy and subsequent treatment with vehicle, OCA, or INT-787 for 8 weeks. Hepatic steatosis, inflammation, and fibrosis (liver lipids, galectin-3, and collagen 1a1 [Col1a1], respectively), as well as plasma alanine transaminase (ALT) and aspartate transaminase (AST) levels, were assessed. Hepatic gene expression was assessed in Lep^*ob/ob*^ mice that were fed the AMLN diet for 14 weeks then treated with vehicle, OCA, or INT-787 for 2 weeks. INT-787, which is equipotent to OCA but more hydrophilic, significantly reduced liver lipids, galectin-3, and Col1a1 compared with vehicle, and to a greater extent than OCA. INT-787 significantly reduced plasma ALT and AST levels, whereas OCA did not. INT-787 modulated a substantially greater number of genes associated with FXR signaling, lipid metabolism, and stellate cell activation relative to OCA in hepatic tissue. These findings demonstrate greater efficacy of INT-787 treatment compared with OCA in improving liver histopathology, decreasing liver enzyme levels, and enhancing gene regulation, suggesting superior clinical potential of INT-787 for the treatment of NASH and other chronic liver diseases.

## Introduction

Nonalcoholic steatohepatitis (NASH) is a progressive form of nonalcoholic fatty liver disease (NAFLD), characterized by hepatic steatosis, hepatocyte injury (ballooning), inflammation, and fibrosis that may lead to cirrhosis and hepatocellular carcinoma [1–3]. In 2020, NASH was estimated to affect up to 6% of the US population, and the number of patients is expected to increase by 63% from 16.5 million cases in 2015 to 27 million in 2030 [3–5]. More than 20% of patients with NASH develop cirrhosis in their lifetime, leading to end-stage liver disease and the need for liver transplant [3,6]. Currently, there are no US Food and Drug Administration–approved pharmacologic therapies for NASH. Available treatments include lifestyle modifications such as diet, exercise, and, in some cases, bariatric surgery [7]. However, lifestyle interventions require sustained weight loss and are usually insufficient for patients with advanced disease and liver fibrosis, highlighting the need for effective pharmacotherapy.

Multiple preclinical and clinical studies support the farnesoid X receptor (FXR) as a promising therapeutic target in NASH [8,9]. FXR, which is highly expressed in the liver and intestine, binds bile acids and plays a vital role in regulating bile acid homeostasis. In addition, FXR regulates lipid and glucose metabolism and inhibits inflammation and fibrosis [8]. Several clinical studies have confirmed that FXR activation leads to reduced inflammation and fibrosis in patients with chronic liver diseases, including primary biliary cholangitis and NASH [10–13]. Based on these positive data, a variety of FXR agonists have been developed for NASH treatment in an attempt to enhance efficacy and reduce side effects using bile acids, steroidal nonbile acids, or nonsteroidal scaffolds [14,15].

The FXR agonists obeticholic acid (OCA) and INT-787 are modified semi-synthetic bile acids derived from the natural FXR agonist chenodeoxycholic acid (CDCA) [16]. OCA is characterized by the insertion of an ethyl group at the C6α position of CDCA, resulting in 100-fold greater potency at FXR (EC_50_ = 0.15 μmol/L) compared with CDCA while also preserving weak potency at the Takeda G protein–coupled receptor 5 (TGR5) receptor (EC_50_ = 15 μmol/L) [16]. INT-787 is a novel OCA derivative hydroxylated at position C11β, resulting in a compound equipotent to OCA at FXR (EC_50_ = 0.14 μmol/L) but with no TGR5 agonistic activity, making INT-787 highly selective for FXR [16]. The additional hydroxyl group also confers differentiated physicochemical properties more similar to hydrophilic bile acids such as ursodeoxycholic acid [16]. Both OCA and INT-787 have been shown previously to be effective in FXR target engagement and to reach the liver and circulating blood [16]. As a bile acid derivative, INT-787 exhibits an enterohepatic profile of exposure similar to endogenous bile acids [16]. In a mouse model of obstructed bile acid flow, FXR activation by INT-787 led to significant reduction of bile acid pool size in both serum and bile, shifted the bile acid pool to a more hydrophilic composition, and prevented damage to the intestinal barrier likely through remodeling of the intestinal microbiome [17].

In 2 clinical trials in patients with NASH, OCA treatment improved fibrosis and prevented progression of NASH. In the phase 2 FLINT trial, significantly more patients in the OCA 25-mg treatment group showed improvements in fibrosis, hepatocellular ballooning, steatosis, and lobular inflammation compared with placebo [12]. In the phase 3 REGENERATE trial, OCA 25 mg significantly improved lobular inflammation, hepatocellular ballooning, and fibrosis in twice as many patients as placebo and prevented progression of fibrotic disease [11,18]. In addition, treatment with OCA reduced alanine transaminase (ALT), aspartate transaminase (AST), gamma-glutamyl transferase, fibrosis-4 score, and FibroTest scores compared with placebo. Patients treated with OCA also exhibited reduced liver stiffness, as assessed by vibration-controlled transient elastography at month 18, compared with those who received placebo [13]. Although multiple FXR agonists have demonstrated histologic improvements in preclinical models of NASH, and OCA has demonstrated antifibrotic activity in a phase 3 study of patients with NASH who had precirrhotic liver fibrosis [11], no data are available about the effects of INT-787 in this regard.

Preclinical studies have demonstrated the clinical translatability of genetically obese (leptin-deficient [Lep^*ob/ob*^]) mice fed a high-fat diet and the utility of this model for investigating pharmacotherapies for NASH [19–24]. High-caloric diets supplemented with fat, carbohydrates (fructose and sucrose), and cholesterol, such as the Amylin liver NASH (AMLN) diet, have previously been used in NASH mouse models [19,22,25,26]. Lep^*ob/ob*^ mice fed the AMLN diet for 12 to 26 weeks display increased levels of plasma cholesterol, ALT, and AST and develop hepatic steatosis and fibrosis typical of NASH, compared with mice fed standard chow [22].

The objective of the present study was to compare the effects of OCA with INT-787 on plasma biomarkers, quantitative histologic disease parameters, and hepatic gene expression profiles in the AMLN diet–induced and biopsy-confirmed mouse model of NASH.

## Materials and methods

### Animals

All animal experiments were conducted in accordance with the internationally accepted principles for the care and use of laboratory animals (license numbers: 2013-15-2934-00784 and 2015-15-0201-00518 issued by the Danish Committee for Animal Research). Male B6.V-Lep^*ob*^/JRj mice (5 weeks old) were obtained from Janvier Labs. Each animal was uniquely identified by an implantable microchip (Pet ID Microchip, E-vet). Mice were fed the AMLN diet, which has been previously described (40% fat [18% trans fat], 20% fructose, and 2% cholesterol) [19,21,22], for 14 or 15 weeks prior to treatment start and during treatment.

### Baseline liver biopsy

Liver biopsy for baseline quantification of fibrosis was performed at pretreatment week −3 (11–12 weeks after initiation of the AMLN diet). Mice were anesthetized using inhaled isoflurane (2%–3%). A small abdominal incision was made in the midline to expose the left lateral lobe of the liver. A cone shaped wedge of liver tissue (~50 mg) was excised from the distal portion of the lobe and fixated in 10% neutral buffered formalin (4% formaldehyde) for histology. The cut surface of the liver was instantly electrocoagulated using bipolar coagulation (ERBE VIO 100 electrosurgical unit). The liver was returned to the abdominal cavity, the abdominal wall sutured, and the skin closed with staples. For postoperative recovery, mice received 5 mg/kg subcutaneous carprofen on the day of the operation and postoperative days 1 and 2.

### Drug treatment

**Fig 1:**
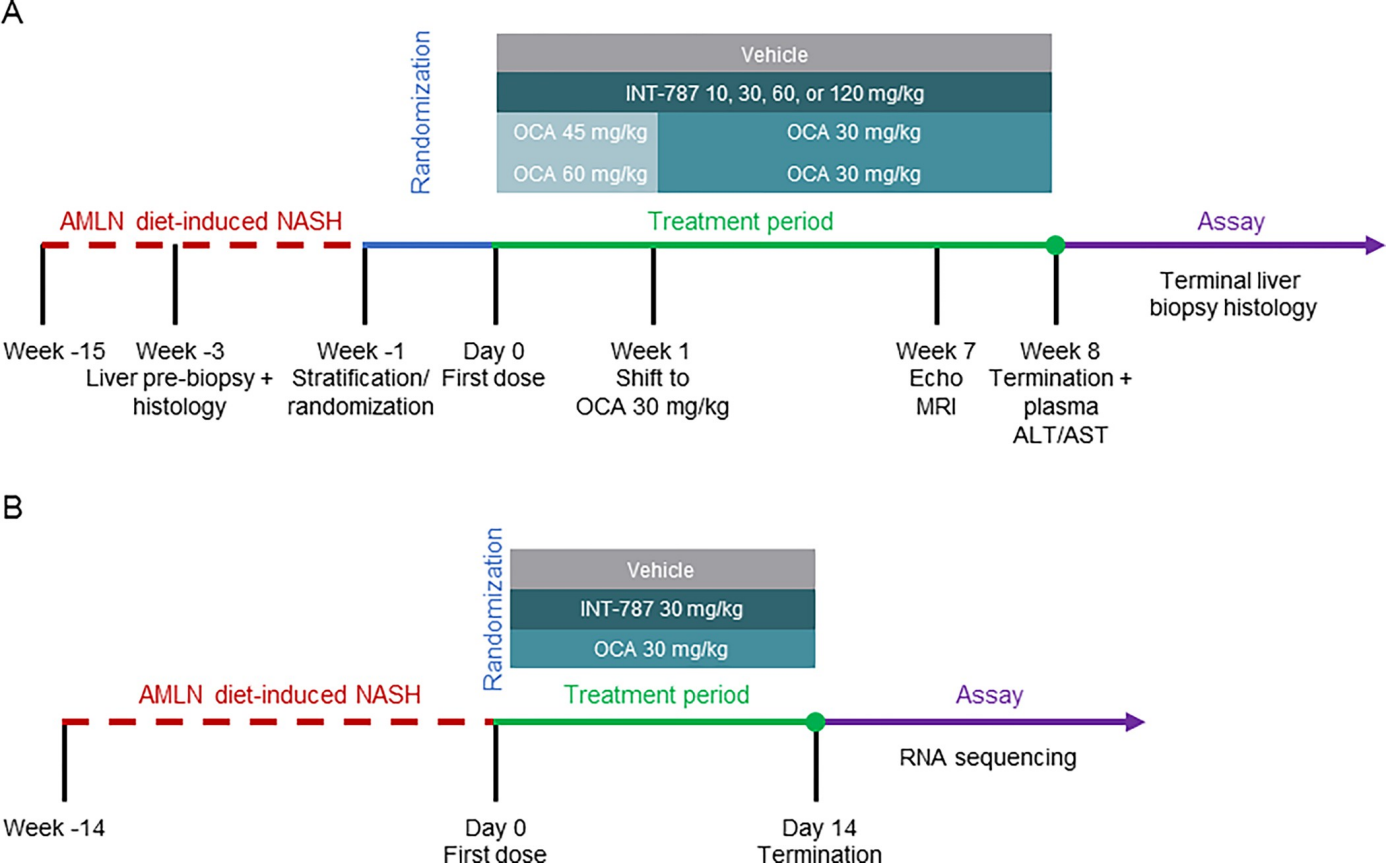
Experimental design for disease progression analysis (A) and mRNA sequencing (B). ALT indicates alanine transaminase; AMLN, Amylin liver NASH; AST, aspartate transaminase; MRI, magnetic resonance imaging; NASH, nonalcoholic steatohepatitis; OCA, obeticholic acid; RNA, ribonucleic acid.

For disease progression analysis, a stratified randomization was used to assign animals into treatment groups (n = 12 per group) based on mean fibrosis as assessed by liver Col1a1 at pretreatment liver biopsy (Fig 1). After 15 weeks on the AMLN diet, treatments were administered orally once daily for 8 weeks with vehicle (0.5% carboxymethylcellulose), OCA (45/30 or 60/30 mg/kg), or INT-787 (10, 30, 60, or 120 mg/kg). In the OCA 45/30-mg/kg and OCA 60/30-mg/kg groups, mice were treated with OCA 45 mg/kg or 60 mg/kg, respectively, for 1 week followed by 30 mg/kg for 7 weeks to avoid potential toxicity.

The disease progression study was conducted to understand the differences between the 2 drugs (OCA and INT-787) on biochemical and histologic endpoints. Once differential effects were found, a second study was conducted to characterize mechanisms of action of the drugs at the gene expression level. For gene expression analysis, stratified randomization into treatment groups (n = 10 per group) was based on body weight. After 14 weeks on the AMLN diet, treatments were administered orally once daily for 2 weeks with vehicle, OCA (30 mg/kg), or INT-787 (30 mg/kg). Pilot experiments demonstrated that treatment for 2 weeks was sufficient to elicit gene expression changes before histologic changes were observed; thus, this timeframe was selected for drug comparison in the second study. The dose chosen for the gene expression experiment was the optimal dose of 30 mg/kg/d for OCA as previously published [21]. A matched dose of INT-787 (30 mg/kg/d) was used for comparison.

### Histopathology assessment

In a few mice, severe liver disease progression was observed, resulting in mortality or necessitating euthanasia (cardiac puncture under isoflurane anesthesia). There was an equal distribution of preterminated and dead animals in each treatment group (INT-787 and OCA) for each dose and in the vehicle group, suggesting that this was not related to drug treatment. For animals that survived through end of study, tissue from the left lateral lobe of the liver (100–200 g) was collected and fixated for histology. One tissue section from the liver biopsy sample for each histologic parameter was used for quantification. Quantitative histologic measures, including liver lipids (steatosis), inflammation biomarker galectin-3, and fibrosis biomarker Col1a1, were assessed at pretreatment and after the 8-week treatment period. Slides with paraffin-embedded sections were deparaffinized in xylene and rehydrated in a series of graded ethanol. For hematoxylin and eosin (H&E) staining, slides were incubated in Mayer’s Hematoxylin (Dako), washed in tap water, stained in Eosin Y solution (Sigma-Aldrich), hydrated, mounted with Pertex, and allowed to dry before scanning. Steatosis detected on H&E-stained slides and immunohistochemistry (IHC)–positive staining were quantified by image analysis using the Visiomorph software (Visiopharm). Quantitative estimates of steatosis and IHC-positive staining (described in more detail below) were calculated as area fractions. Galectin-3 (Biolegend, Cat. 125402) and Col1a1 (Southern Biotech, Cat. 1310–01) IHC staining were performed using standard procedures. Briefly, after antigen retrieval and blocking of endogenous peroxidase activity, slides were incubated with primary antibody. For Col1a1 staining, the primary antibody was detected using biotinylated secondary antibody and amplified using a vectastain-tyramide signal amplification-vectastain method, a polymeric horseradish peroxidase (HRP)–Iinker antibody conjugate. For galectin-3 staining, the primary antibody was detected using a linker secondary antibody followed by amplification using a polymeric HRP-Iinker antibody conjugate. For both assessments, the primary antibody was visualized with 3,3’-diaminobenzidine as chromogen. Sections were counterstained in hematoxylin and mounted with coverslips.

### Liver enzyme analysis

After the 8-week treatment, terminal blood samples were assayed for plasma concentrations of ALT and AST, which were measured using commercial kits (Roche Diagnostics) on the Cobas C-501 autoanalyzer according to the manufacturer’s instructions.

### mRNA sequencing

After the 2-week treatment, animals were sacrificed for mRNA sequencing from terminal liver samples (Fig 1). RNA was quantified using Qubit (Thermo Fisher Scientific). The RNA quality was determined using a bioanalyzer with RNA 6000 Nano kit (Agilent). RNA sequence libraries were prepared with NeoPrep (Illumina) using Illumina TruSeq stranded mRNA Library kit for NeoPrep and sequenced on the NextSeq 500 (Illumina) with NSQ 500 hi-Output KT v2 (75 CYS, Illumina). Reads were aligned to the Genome Reference Consortium Mouse Build 38 (GRCm38) version 89 Ensembl *Mus musculus* genome using Spliced Transcripts Alignment to a Reference (STAR) version 2.5.2a with default parameters [27]. Differential gene expression analysis was carried out using DESeq2 in R programming [28].

### Statistical analysis

Plasma levels of ALT and AST and the percentage of fractional area for liver lipids, galectin-3, and Col1a1 were summarized via descriptive statistics. Differences between OCA or INT-787 treatment groups with vehicle were assessed via 1-way analysis of variance (ANOVA) with Dunnett post hoc tests at the 0.05 significance level. Comparisons between OCA and INT-787 groups were assessed by 1-way ANOVA and Bonferroni’s post hoc test at the significance level of 0.05. Regulated genes with a Benjamini-Hochberg adjusted *p* ≤ 0.05 (5% false discovery rate) were regarded as statistically significant. The Reactome pathway database was used for gene annotation in gene set analysis using the R package PIANO version 1.18.1, with the Stouffer method and Benjamini-Hochberg adjusted *p* values (false discovery rate < 0.01).

## Results

### Effects of INT-787 and OCA on histologic and biochemical NASH progression

**Fig 2:**
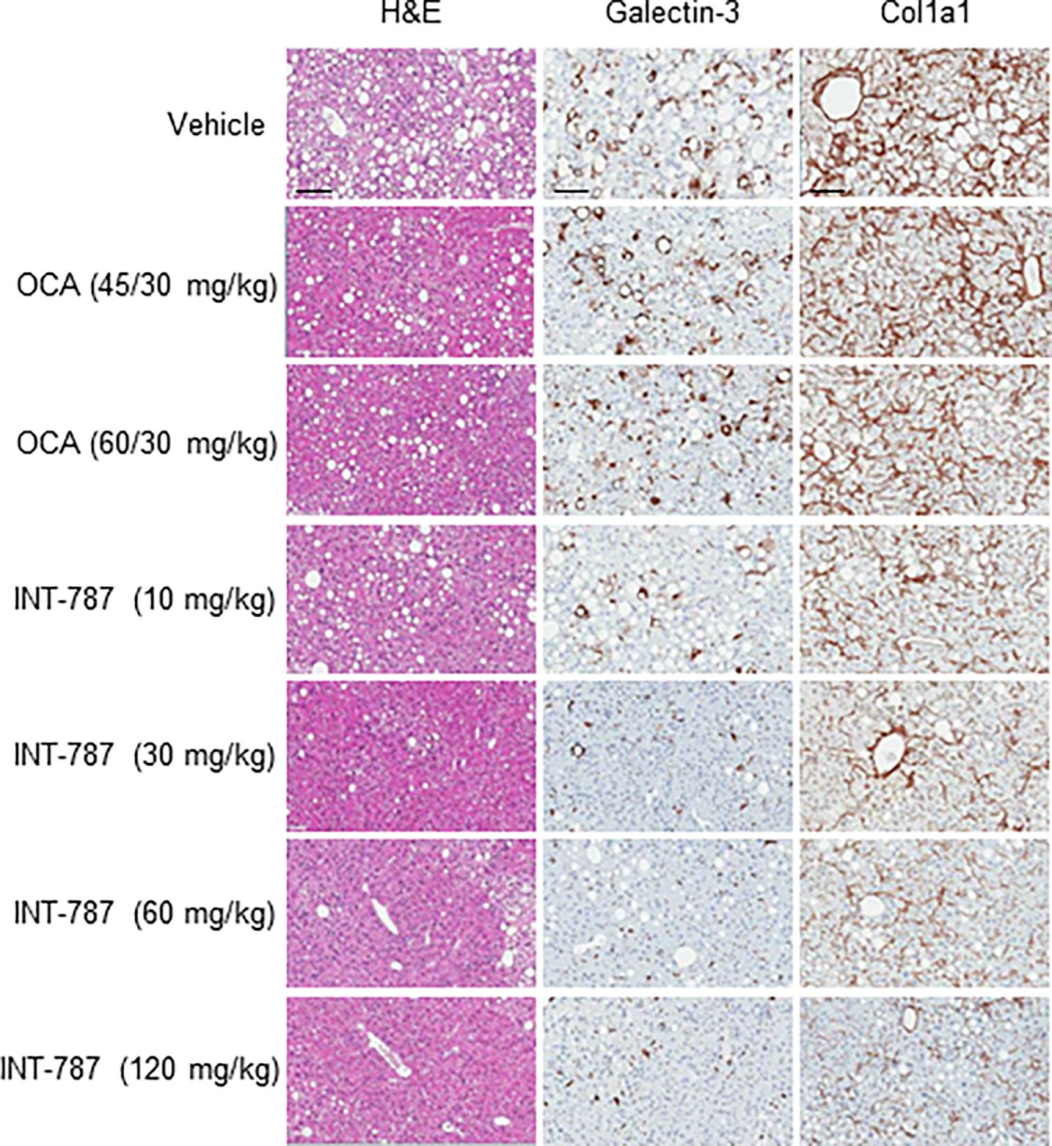
Representative images of liver histopathologic assessments of steatosis (H&E staining), inflammation (galectin-3 staining), and fibrosis (Col1a1 staining). Col1a1 indicates collagen type 1 alpha 1 chain; H&E, hematoxylin and eosin; OCA, obeticholic acid. Stains are magnified 20×, scale bar = 100 μm.

**Fig 3:**
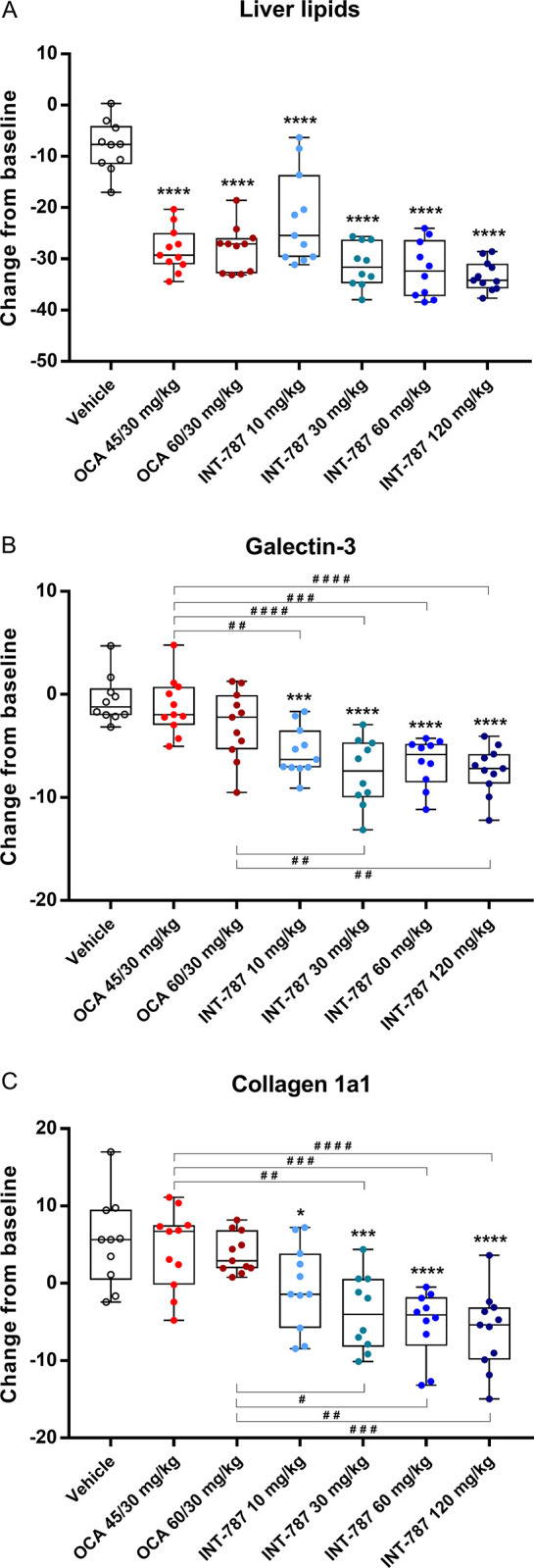
Levels of histopathology markers quantified by percentage fractional area for steatosis (A), inflammation (B), and fibrosis (C). Data are percentage of fractional area change from baseline with minimum, maximum, and median values and interquartile range (Q1 and Q3). *p < 0.05, ***p < 0.001, ****p < 0.0001 compared with vehicle by 1-way analysis of variance with Dunnett post hoc test. ^#^*p* < 0.05, ^##^*p* < 0.01, ^###^*p* < 0.001, ^####^*p* < 0.0001 by 1-way analysis of variance with Bonferroni post hoc test. Col1a1 indicates collagen type 1 alpha 1 chain; OCA indicates obeticholic acid. Vehicle, n = 10; OCA 45/30 mg/kg, n = 11; OCA 60/30 mg/kg, n = 11; INT-787 10 mg/kg, n = 11; INT-787 30 mg/kg, n = 10; INT-787 60 mg/kg, n = 10; INT-787 120 mg/kg, n = 11.

After 8 weeks of treatment, both OCA and INT-787 significantly reduced liver steatosis at all doses tested compared with vehicle in mice fed the AMLN diet for 15 weeks (Figs 2 and 3). INT-787 30 to 120 mg/kg reduced liver lipids (steatosis) more than both OCA treatment groups. A 74% reduction in steatosis was observed after INT-787 30-mg/kg treatment, whereas steatosis was decreased by 61% in both OCA treatment groups compared with vehicle. No significant differences in steatosis were observed between any doses of INT-787 and either dose of OCA. Terminal liver weight (grams) was significantly reduced by 26% and 27% after OCA 45/30-mg/kg and 60/30-mg/kg treatment (both doses, *p* < 0.001), respectively, compared with vehicle. INT-787 10 mg/kg, 30 mg/kg, and 60 mg/kg also significantly reduced terminal liver weight by 24% (*p* < 0.01), 27% (*p* < 0.001), and 23% (*p* < 0.01), respectively, whereas no significant differences in terminal weight were observed between INT-787 120 mg/kg and vehicle.

All doses of INT-787 significantly reduced inflammation marker galectin-3 compared with vehicle, whereas no significant differences were observed between OCA treatment and vehicle (Figs 2 and 3). Significant decreases in galectin-3 were observed with all doses of INT-787 compared with OCA 45/30 mg/kg. Galectin-3 significantly decreased with INT-787 30 mg/kg and 120 mg/kg compared with OCA 60/30 mg/kg. Notably, a 64% reduction in galectin-3 was observed after INT-787 30-mg/kg treatment, whereas a comparable dose of OCA did not significantly reduce galectin-3 compared with vehicle.

INT-787 treatment induced significant dose-dependent reductions of liver Col1a1, a major contributor to hepatic fibrosis (Figs 2 and 3) [20]. A 44% reduction in Col1a1 was observed after INT-787 30-mg/kg treatment, whereas a comparable dose of OCA was ineffective compared with vehicle. INT-787 30 to 120 mg/kg significantly reduced Col1a1 compared with OCA 45/30 and 60/30 mg/kg.

**Fig 4:**
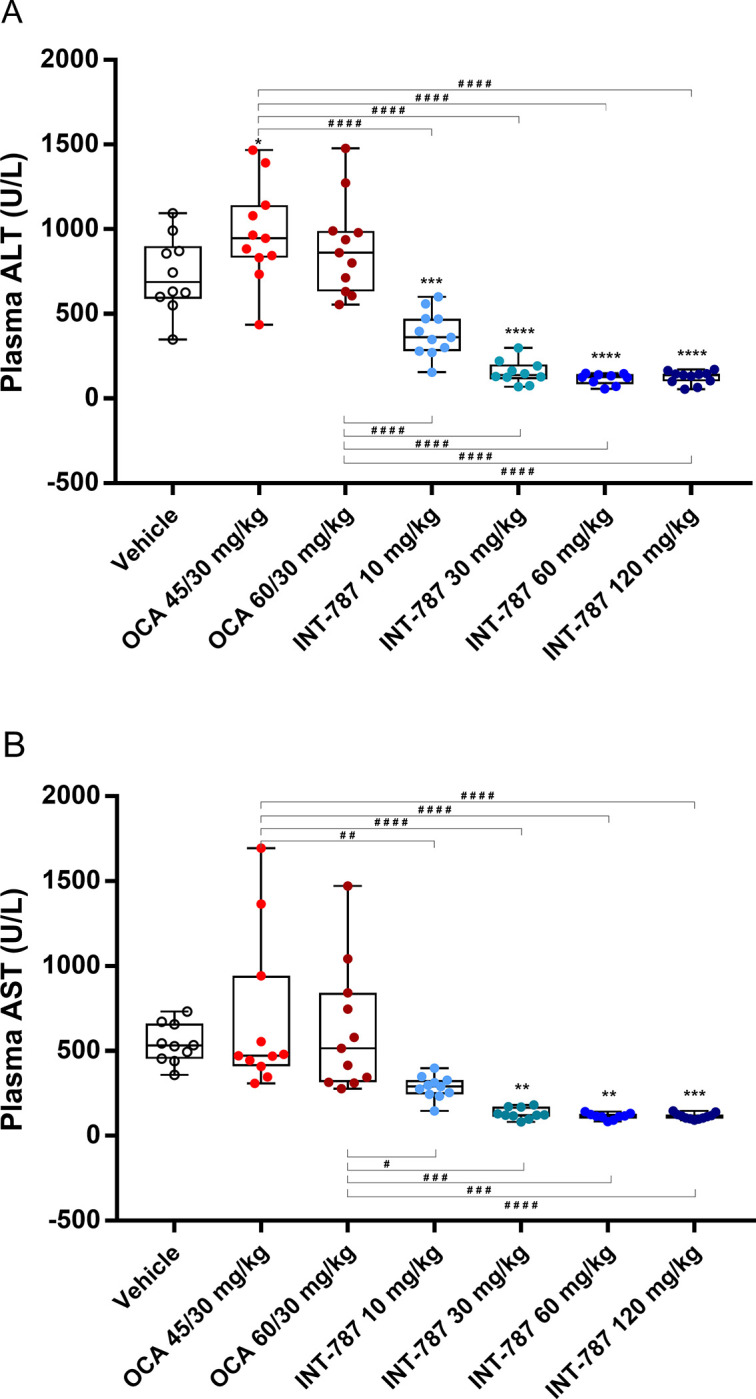
Levels of the plasma liver enzymes ALT (A) and AST (B) at termination. Data are individual plasma ALT and AST (U/L) values; minimum, maximum, and median values; and interquartile range (Q1 and Q3). *p < 0.05, **p < 0.01, ***p < 0.001, ****p < 0.0001 compared with vehicle by 1-way analysis of variance with Dunnett post hoc test. ^#^*p* < 0.05, ^##^*p* < 0.01, ^###^*p* < 0.001, ^####^*p* < 0.0001 by 1-way analysis of variance with Bonferroni post hoc test. ALT indicates alanine transaminase; AST, aspartate transaminase; OCA, obeticholic acid. Vehicle, n = 10; OCA 45/30 mg/kg, n = 11; OCA 60/30 mg/kg, n = 11; INT-787 10 mg/kg, n = 11; INT-787 30 mg/kg, n = 10; INT-787 60 mg/kg, n = 9; INT-787 120 mg/kg, n = 11 for animals with plasma ALT and AST measurements at termination.

In addition, INT-787 significantly reduced plasma ALT levels (at all doses) and AST levels (at 30–120 mg/kg) compared with vehicle, whereas no significant differences were observed between OCA treatment and vehicle (Fig 4). Improvements in plasma liver enzyme levels were similar with the 3 highest doses of INT-787 (30, 60, and 120 mg/kg). All doses of INT-787 significantly reduced plasma ALT levels compared with OCA 45/30 mg/kg and 60/30 mg/kg. Significant decreases in plasma AST levels were observed with each INT-787 dose compared with OCA 45/30 mg/kg and 60 mg/kg.

**Fig 5:**
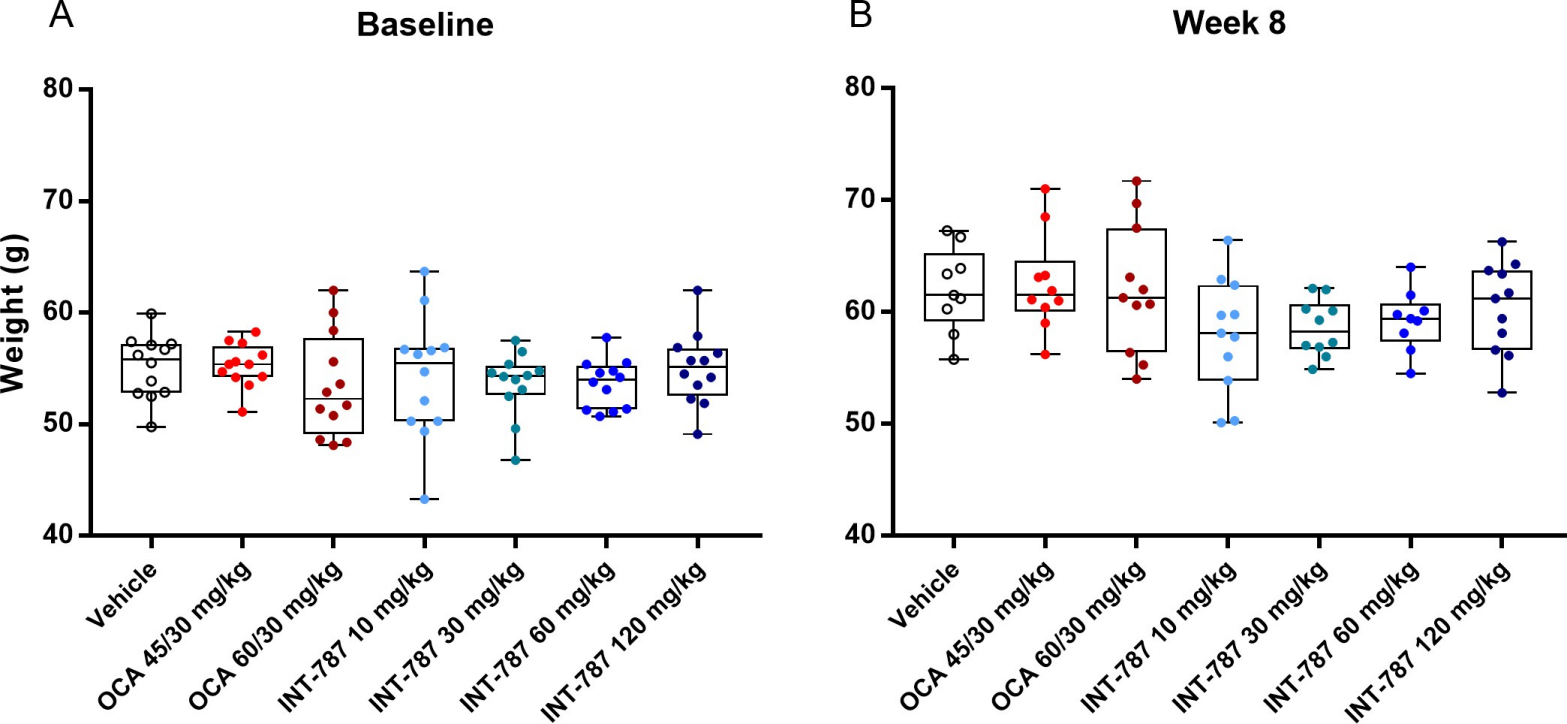
Body weight (g) at baseline (A) and end of study (week 8) (B). Data are individual body weights; minimum, maximum, and median values; and interquartile range (Q1 and Q3). OCA indicates obeticholic acid. Vehicle, n = 9; OCA 45/30 mg/kg, n = 10; OCA 60/30 mg/kg, n = 11; INT-787 10 mg/kg, n = 11; INT-787 30 mg/kg, n = 10; INT-787 60 mg/kg, n = 9; INT-787 120 mg/kg, n = 11 for animals with body weight measurements at day 57.

No significant differences in body weight were observed after OCA and INT-787 treatment compared with vehicle (Figs 5 and S1), and no significant differences in lean tissue mass were observed after treatment by any dose of either drug compared with vehicle. There were no significant differences in lean tissue mass observed between any INT-787 doses and either OCA dose. Similarly, no significant differences in fat tissue mass were observed after treatment with any dose of either drug compared with vehicle. Significant differences in fat tissue mass were only observed between OCA 45/30 mg/kg and INT-787 60 mg/kg (*p* < 0.05), and no significant differences were observed with any other OCA and INT-787 dose comparisons.

### Effects of INT-787 and OCA on hepatic gene expression in a biopsy-proven NASH model

**Fig 6:**
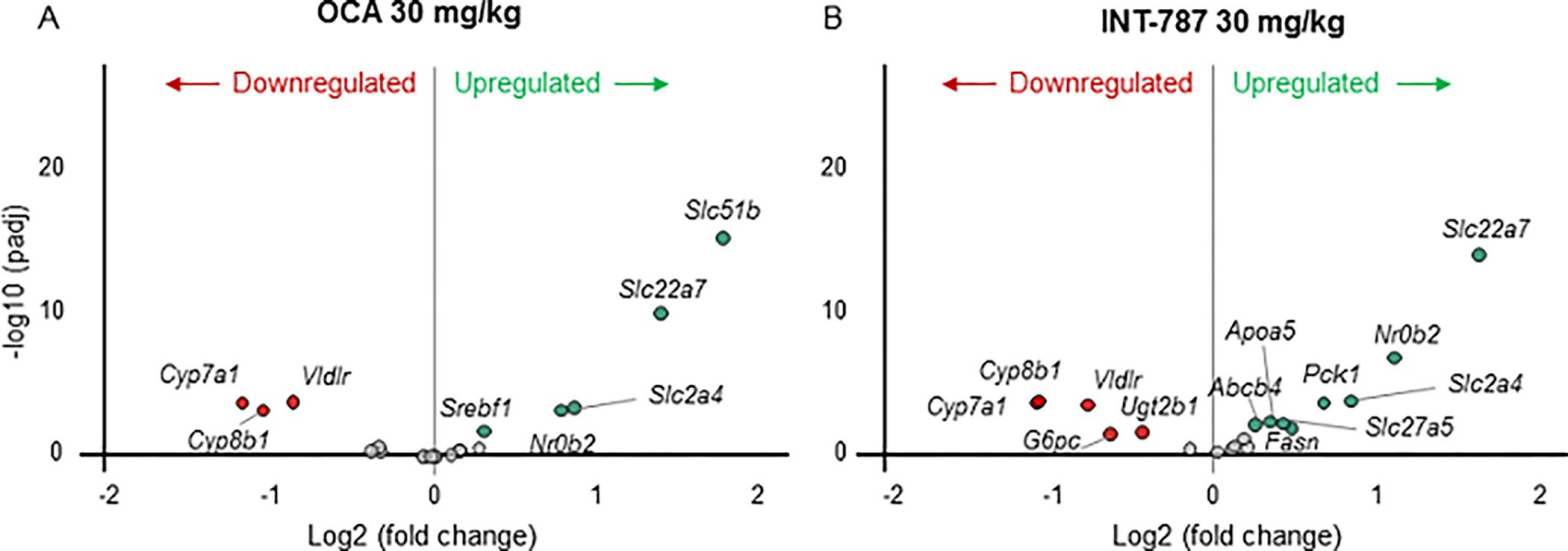
Genes associated with farnesoid X receptor signaling in the liver significantly up- or downregulated after treatment with OCA 30 mg/kg or INT-787 30 mg/kg compared with vehicle. Differences in expression levels of genes meeting the adjusted *p* < 0.05 threshold for significance are shown in red or green. Gray circles represent genes in the farnesoid X receptor signaling pathway that were not significantly up- or downregulated after treatment. OCA indicates obeticholic acid; padj, adjusted *p* value. Vehicle, n = 10; OCA 30 mg/kg, n = 9; INT-787 30 mg/kg, n = 9.

**Fig 7:**
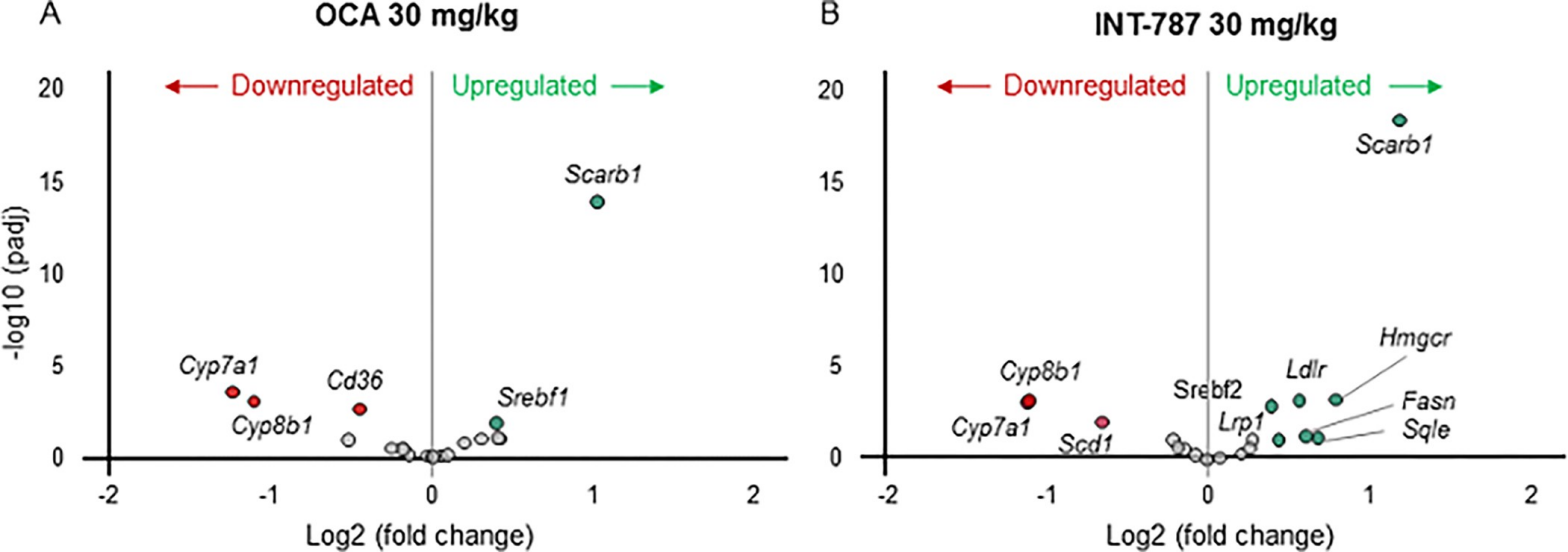
Genes associated with lipid metabolism in the liver significantly up- or downregulated after treatment with OCA 30 mg/kg or INT-787 30 mg/kg compared with vehicle. Differences in expression levels of genes meeting the adjusted *p* < 0.05 threshold for significance are shown in red or green. Gray circles represent genes in the lipid metabolism pathway that were not significantly up- or downregulated after treatment. OCA indicates obeticholic acid; padj, adjusted *p* value. Vehicle, n = 10; OCA 30 mg/kg, n = 9; INT-787 30 mg/kg, n = 9.

**Fig 8:**
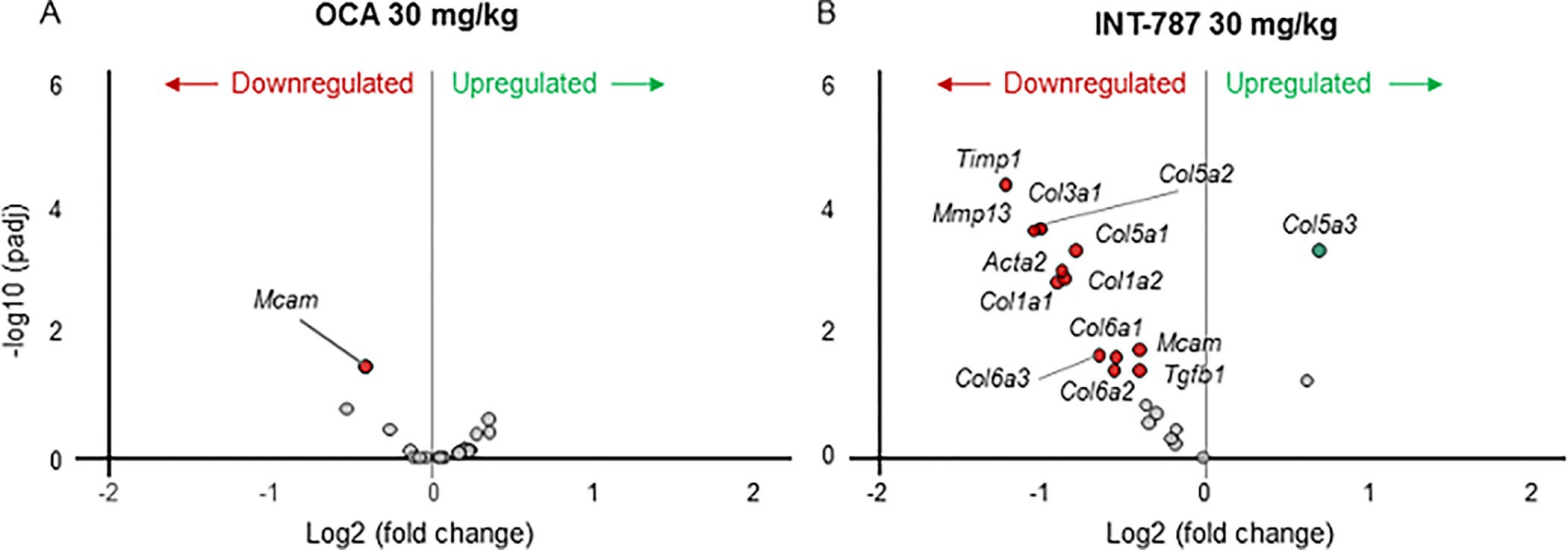
Genes associated with stellate cell activation (fibrosis) in the liver significantly up- or downregulated after treatment with OCA 30 mg/kg or INT-787 30 mg/kg compared with vehicle. Differences in expression levels of genes meeting the adjusted *p* < 0.05 threshold for significance are shown in red or green. Gray circles represent genes in the stellate cell activation pathway that were not significantly up- or downregulated after treatment. OCA indicates obeticholic acid; padj, adjusted *p* value. Vehicle, n = 10; OCA 30 mg/kg, n = 9; INT-787 30 mg/kg, n = 9.

After 2 weeks of treatment, INT-787 30 mg/kg significantly modulated 2063 differentially expressed genes compared with 728 genes modulated by OCA 30 mg/kg in hepatic tissue of mice with biopsy-proven NASH. Compared with vehicle, INT-787 significantly modulated the expression of a greater number of FXR signaling–related genes associated with lipid transport into bile (*Abcb4)*, bile acid glucuronidation (*Ugt2b1*) and reconjugation (*Slc27a5*), triglyceride synthesis (*Fasn*), circulatory lipid transport (*Apoa5*), glucose homeostasis (*Pck1*), and glucose synthesis (*G6pc*) than OCA (Fig 6). A greater number of genes associated with regulation of triglyceride synthesis (*Fasn*, *Scd1*), cholesterol uptake (*Ldlr*, *Lrp1*), and cholesterol synthesis (*Srebf2*, *Hmgcr*, *Sqle*) were significantly modulated by INT-787 than by OCA (Fig 7). INT-787 also significantly downregulated the expression of a greater number of fibrosis-related genes than OCA, including those associated with myofibroblast motility (*Acta2*) and proliferation (*Tgfβ1*), fibrogenesis (*Col1a1*, *Col1a2*, *Col3a1*, *Col5a1*, *Col5a2*, *Col6a1*, *Col6a2*, *Col6a3*), and extracellular matrix (ECM) turnover (*Timp1*, *Mmp13*) (Fig 8).

## Discussion

This study rigorously compared multiple doses of the FXR agonists OCA and INT-787 on plasma biomarkers, quantitative histologic disease parameters, and hepatic gene expression profiles in the AMLN diet–induced and biopsy-confirmed mouse model of NASH. Overall, INT-787 elicited greater improvements in steatosis, inflammation, and fibrosis relative to OCA. All INT-787 doses (except 10 mg/kg) reduced liver steatosis to a greater extent than OCA. INT-787 significantly reduced expression of galectin-3 and Col1a1 (markers of inflammation and fibrosis, respectively) at all doses whereas OCA did not. Further, levels of plasma liver enzymes (ALT and AST) were significantly reduced by INT-787 but not by OCA.

The differentiation of INT-787 compared with OCA is also evidenced by the modulation of 3-fold greater number of genes in the liver. These changes suggest that INT-787 more robustly activates FXR relative to OCA, as increased changes within classical genes associated with FXR signaling, bile acid regulation, lipid metabolism, and fibrosis were noted. These gene expression modifications also include those involved with bile acid import/export (i.e., *Abcb4*) and bile acid re-conjugation (i.e., *Slc27a5)*. Bile acids play a pivotal role in regulation of insulin signaling, glucose metabolism, and metabolic homeostasis [29,30]. Dysregulation of bile acids can be a consequence of NASH but can also lead to inflammation and NASH progression [31,32]. Compared with OCA, FXR activation by INT-787 induces higher levels of *Shp* expression, which inhibits bile acid synthesis by targeting *Cyp7a1* [33]. INT-787 also significantly increased expression of *Pck1*, consistent with the observation that FXR activation modulates expression of *Pck1* to regulate glucose homeostasis [34,35]. In addition, INT-787 induced hepatic expression of *Apoa5*, which activates lipoprotein lipase [36], an enzyme involved in degradation of circulating triglycerides [37]. INT-787 upregulated the expression *Ldlr*, a gene associated with cholesterol uptake and that is downregulated in patients with NAFLD/NASH [38]. Finally, INT-787 decreased expression of *Scd1*, a gene involved in regulation of triglyceride synthesis that is increased in patients with NAFLD and in *ob/ob* mice on a high-fat diet [39].

Histopathologic reduction of fibrosis is generally associated with decreases in multiple components of the ECM at the mRNA and protein level [40,41]. The Col1a1 isoform is a key fibrillar collagen involved in liver fibrosis and both *Col1a1* mRNA and protein were reduced by INT-787 treatment. Other genes involved in fibrosis that were significantly downregulated by INT-787 include *Col1a2*, *Col3a1*, *Col5a1*, *Col5a2*, *Col6a1*, *Col6a2*, *Col6a3*, *Tgfb1*, *Acta2*, and *Timp1*. Transforming growth factor (TGF)-β1 (encoded by *Tgfb1*) regulates fibroblast proliferation, migration, activation, and differentiation [42] as well as hepatic stellate cell (HSC) activation, which drive induction and progression of fibrosis [43]. Activation of HSCs results in α-smooth muscle actin (encoded by *Acta2*) expression and deposition of fibrous ECM [43]. *Timp1* plays an important role in ECM turnover associated with fibrosis [40]. Given the key role of activated HSCs in the development and progression of fibrosis, treatments targeting activated HSCs are critical for antifibrotic therapy [43]. Our findings indicate that all these genes associated with HSC activation were significantly downregulated by INT-787 but not by OCA. A recent study has shown a greater effect of INT-787 compared with OCA on restoration of Reck expression in *ob/ob* NASH mice fed a high-fat diet. Reck is a key regulator of metalloproteases and inhibitor of Adam10 and Adam17, which are involved in inflammation and fibrogenesis [44]. Collectively, these findings support superior therapeutic effects of INT-787 compared with OCA on metabolic regulation and inhibition of inflammation and fibrosis in the treatment of NASH.

Previous target engagement studies using quantitative reverse transcription polymerase chain reaction assays in HepG2 cells assessed gene expression after treatment with INT-787 or OCA [16]. INT-787 and OCA modulated expression of 3 FXR target genes—*Cyp7a1*, *Shp*, and *OST-α*—at similar levels, but INT-787 induced greater expression of *Bsep* [16]. Findings from in vivo studies showed that INT-787 induced higher expression of ileal fibroblast growth factor 15 (*Fgf15*) compared with OCA, leading to repression of *Cyp7a1*, which encodes the rate-limiting enzyme of bile acid synthesis [16]. The differential gene modulation by INT-787 may depend, at least in part, on the insertion of a hydroxy group at the C11β position, enabling high specificity at the FXR receptor [16]. This subtle change in chemical structure may also cause differential binding to FXR and activation of distinct signaling pathways, which may contribute to explaining the gene regulation patterns observed in our study and warrants further investigation. Differential glyco- and tauro-conjugate formation or activity and differences in pharmacokinetics (e.g., distribution, half-life, hepatic residence time) may also play a role in the differential gene regulation induced by INT-787 compared with OCA.

Initial data from an ongoing phase 1 trial of INT-787 in healthy participants support a favorable safety and tolerability profile [45]. INT-787 has 16-fold higher water solubility than OCA and a higher critical micellular concentration (16.8 vs 2.9 mmol/L), which are associated with lower detergency, resulting in reduced cytotoxicity risk [16]. FXR agonists, including OCA, have been associated with pruritus [46,47]. In patients with NASH, nonbile acid–derived FXR agonists EDP-305 and tropifexor (which is also a nonsteroidal FXR agonist) were also associated with pruritus as the most commonly reported adverse event, suggesting that this is a class effect [48,49]. Similarly, pruritus has also been reported in NASH clinical trials using cilofexor, another nonsteroidal FXR agonist, and nidufexor, a partial FXR agonist, further indicating that pruritus induction is a class effect shared by FXR agonists [47,50–52]. Mild pruritus was also reported in 2 participants from the ongoing phase 1 trial of INT-787 in healthy participants; however, this was observed at lower rather than higher doses [45].

The current preclinical findings suggest that INT-787 may potentially exert greater therapeutic effects in NASH relative to OCA. These included more significant reductions in steatosis and inflammation as well as significant decreases in plasma ALT and AST levels, which support reduction in liver damage. These data provide a foundation for the use of INT-787 in clinical trials as a potentially more effective therapy than OCA to treat NASH. In addition, the beneficial effects of INT-787 on multiple parameters of metabolic regulation, the marked inhibition of liver injury, and the clear anti-inflammatory and antifibrotic effects, coupled with the favorable safety profile of the compound, indicate its potential in the treatment of other chronic liver diseases. For example, NASH and alcohol-associated hepatitis (AH) share common pathogenetic traits, including hepatic inflammation and fibrosis and gut microbiota dysbiosis [53]. Moreover, similar histologic features are observed in both NASH and AH, including steatosis, lobular inflammation, and ballooning [53]. The AMLN diet–induced NASH model used in the current study showed hallmark features of both NASH and AH, including steatosis, inflammation, and fibrosis, which improved with INT-787 treatment, suggesting potential clinical translatability to both diseases. Indeed, INT-787 is currently under evaluation for the treatment of severe AH in the FRESH study (NCT05639543) [54].

In conclusion, based on the significant dose-dependent improvements in liver histopathology, liver enzyme levels, and enhanced gene regulation, these data indicate superior clinical potential of INT-787 compared with OCA for the treatment of NASH and other chronic liver diseases.

## Supporting information

S1 Fig Change in body weight from baseline to week 8. Data are expressed as mean ± SEM. OCA indicates obeticholic acid; SEM, standard error of the mean.
(TIF)

S1 Raw data (XLSX)
