# Supplementary figures and images for: Increased hepatoprotective effects of the novel farnesoid X receptor agonist INT-787 versus obeticholic acid in a mouse model of nonalcoholic steatohepatitis
Source: PLoS One. 2024 Apr 25;19(4):e0300809. doi: 10.1371/journal.pone.0300809 (PMC11045142; doi:10.1371/journal.pone.0300809)

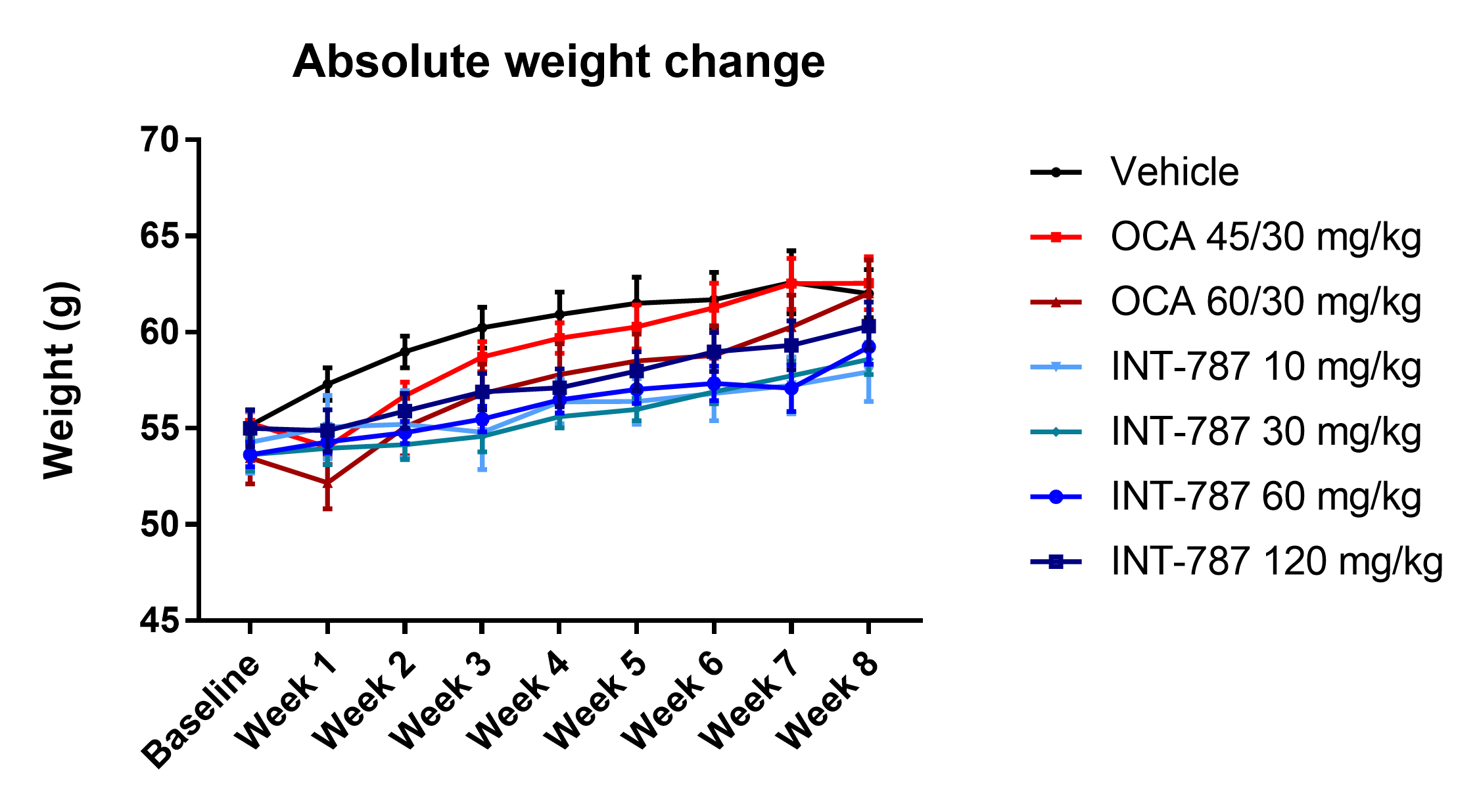

Supplement: S1 Fig — Data are expressed as mean ± SEM. OCA indicates obeticholic acid; SEM, standard error of the mean. (TIF) [file pone.0300809.s001.tif]
